# How New Technologies Can Improve Prediction, Assessment, and Intervention in Obsessive-Compulsive Disorder (e-OCD): Review

**DOI:** 10.2196/11643

**Published:** 2019-12-10

**Authors:** Florian Ferreri, Alexis Bourla, Charles-Siegfried Peretti, Tomoyuki Segawa, Nemat Jaafari, Stéphane Mouchabac

**Affiliations:** 1 Sorbonne Université Department of Adult Psychiatry and Medical Psychology APHP, Saint-Antoine Hospital Paris France; 2 Jeanne d'Arc Hospital INICEA Group Saint Mandé France; 3 INSERM, Pierre Deniker Clinical Research Unit Henri Laborit Hospital & Experimental and Clinical Neuroscience Laboratory Poitiers University Hospital Poitier France

**Keywords:** obsessive-compulsive disorder, ecological momentary assessment, biofeedback, digital biomarkers, digital phenotyping, mobile health, virtual reality, machine learning

## Abstract

**Background:**

New technologies are set to profoundly change the way we understand and manage psychiatric disorders, including obsessive-compulsive disorder (OCD). Developments in imaging and biomarkers, along with medical informatics, may well allow for better assessments and interventions in the future. Recent advances in the concept of digital phenotype, which involves using computerized measurement tools to capture the characteristics of a given psychiatric disorder, is one paradigmatic example.

**Objective:**

The impact of new technologies on health professionals’ practice in OCD care remains to be determined. Recent developments could disrupt not just their clinical practices, but also their beliefs, ethics, and representations, even going so far as to question their professional culture. This study aimed to conduct an extensive review of new technologies in OCD.

**Methods:**

We conducted the review by looking for titles in the PubMed database up to December 2017 that contained the following terms: [Obsessive] AND [Smartphone] OR [phone] OR [Internet] OR [Device] OR [Wearable] OR [Mobile] OR [Machine learning] OR [Artificial] OR [Biofeedback] OR [Neurofeedback] OR [Momentary] OR [Computerized] OR [Heart rate variability] OR [actigraphy] OR [actimetry] OR [digital] OR [virtual reality] OR [Tele] OR [video].

**Results:**

We analyzed 364 articles, of which 62 were included. Our review was divided into 3 parts: prediction, assessment (including diagnosis, screening, and monitoring), and intervention.

**Conclusions:**

The review showed that the place of connected objects, machine learning, and remote monitoring has yet to be defined in OCD. Smartphone assessment apps and the Web Screening Questionnaire demonstrated good sensitivity and adequate specificity for detecting OCD symptoms when compared with a full-length structured clinical interview. The ecological momentary assessment procedure may also represent a worthy addition to the current suite of assessment tools. In the field of intervention, CBT supported by smartphone, internet, or computer may not be more effective than that delivered by a qualified practitioner, but it is easy to use, well accepted by patients, reproducible, and cost-effective. Finally, new technologies are enabling the development of new therapies, including biofeedback and virtual reality, which focus on the learning of coping skills. For them to be used, these tools must be properly explained and tailored to individual physician and patient profiles.

## Introduction

### Background

Obsessive-compulsive disorder (OCD) is a severe and frequent disorder with an estimated lifetime prevalence of 2.3% [1]. It has a poor outcome, with a remission rate of just 53% (95% CI 42‑65) [2]. OCD typically runs a chronic course, with sequential periods of remission and relapse, and is associated with disabling comorbidities, including major depressive disorder (15%), social anxiety disorder (14%), generalized anxiety disorder (13%), persistent depressive disorder (13%), tic disorder (12.5%), body dysmorphic disorder (8.71%), and self-harming behavior (7.43%) [3]. Major functional and emotional impairments are often seen, with an impact on quality of life. In this context, being able to predict the outcome, accurately assess OCD, and intervene in OCD are a major health issue.

New technologies are set to profoundly change our way of practicing psychiatry. At the interface of e-health, new technologies, and clinical observation, a large number of new tools are currently being developed for the assessment and treatment of several psychiatric disorders. Applied to the OCD field, we can talk about “e-OCD” for “e-health technology applied to OCD” just as we can talk about e-PTSD (for posttraumatic stress disorder) or e-Addictology (in the field of addictive disorders). Clinicians currently rely on conventional assessment methods, based on the systematic collection of clinical data during consultations, sometimes using standardized assessment tools (eg, Yale‑Brown Obsessive‑Compulsive Scale, Y-BOCS) [4]. New tools are disrupting this *classical* psychiatry. The recent development of *digital phenotyping* [5], which involves the extraction of psychiatric disorder characteristics by computerized measurement tools via a smartphone or connected device, is one paradigmatic example. Several digital phenotype models are now emerging for schizophrenia [6] and mood disorders [7]. Some behaviors can now be digitally objectified: hyperactivity can be picked up by an accelerometer, manic graphorrhea (a symptom of motor excitement exhibited as continual and incoherent writing) manifests itself in an increase in the number of text messages sent, and even reckless spending can be monitored by a smartphone app. For example, the frequent or excessive hand washing that occurs in some people with OCD could be assessed with a connected wristband.

In OCD, detailed behavioral assessments have been made possible by the ubiquitous use of smartphones to collect large amounts of data that, until recently, were not available to psychiatrists. New methods of data collection can be classified as either active or passive [8]: *active* (or live) data collection refers to all self-assessment procedures that can be implemented on a computer or smartphone (requiring input from the patient); *passive* data collection, via motion detection, smartphone use, and so on, involves background tasks in which patients do not know when data are being collected, thus minimizing the observer’s influence. In the field of care, the expansion of Web-based and smartphone-based interventions holds out the prospect of having a therapist in the pocket [9], and the accessibility of virtual reality (VR) [10] also appears useful in OCD. Machine learning (ML), a special form of artificial intelligence (AI) that classifies data according to a number of variables, allows of patterns to be identified that can then be used to predict treatment outcome [11].

### Objective

Recent reviews of how new technologies can improve prediction, assessment, and intervention in posttraumatic stress disorder (PTSD) [12] or addictive behaviors [13] have shown that, although they have the ability to profoundly change the way we practice psychiatry, the likely impact of these new technologies on health professionals’ practice has yet to be determined [14]. All these innovations, although being at very different stages of development, have the potential to disrupt current practices. To inform health care practitioners about the opportunities and future challenges offered by these new technologies, and their shortcomings, we conducted a review of the technologies that can be used for treatment outcome prediction, assessment, and intervention in OCD.

## Methods

We conducted a review by looking for titles in the PubMed database up to December 2017 that contained the following terms: [Obsessive] AND [Smartphone] OR [phone] OR [Internet] OR [Device] OR [Wearable] OR [Mobile] OR [Machine learning] OR [Artificial] OR [Biofeedback] OR [Neurofeedback] OR [Momentary] OR [Computerized] OR [Heart rate variability] OR [actigraphy] OR [actimetry] OR [digital] OR [virtual reality] OR [Tele] OR [video].

The following inclusion criteria were used to identify studies involving OCD prediction, assessment, and intervention: eHealth apps (ie, computer-, smartphone-, or tablet-based apps), including telemedicine (ie, videoconferencing or phone-delivered cognitive behavioral therapy, CBT); wearable device (ie, smartphone sensor or electrocardiogram, ECG); or machine learning–based categorization.

Exclusion criteria were OCD symptoms not included as a primary or secondary outcome measure and focus on conventional media (ie, television, radio, or telephone).

**Figure 1:**
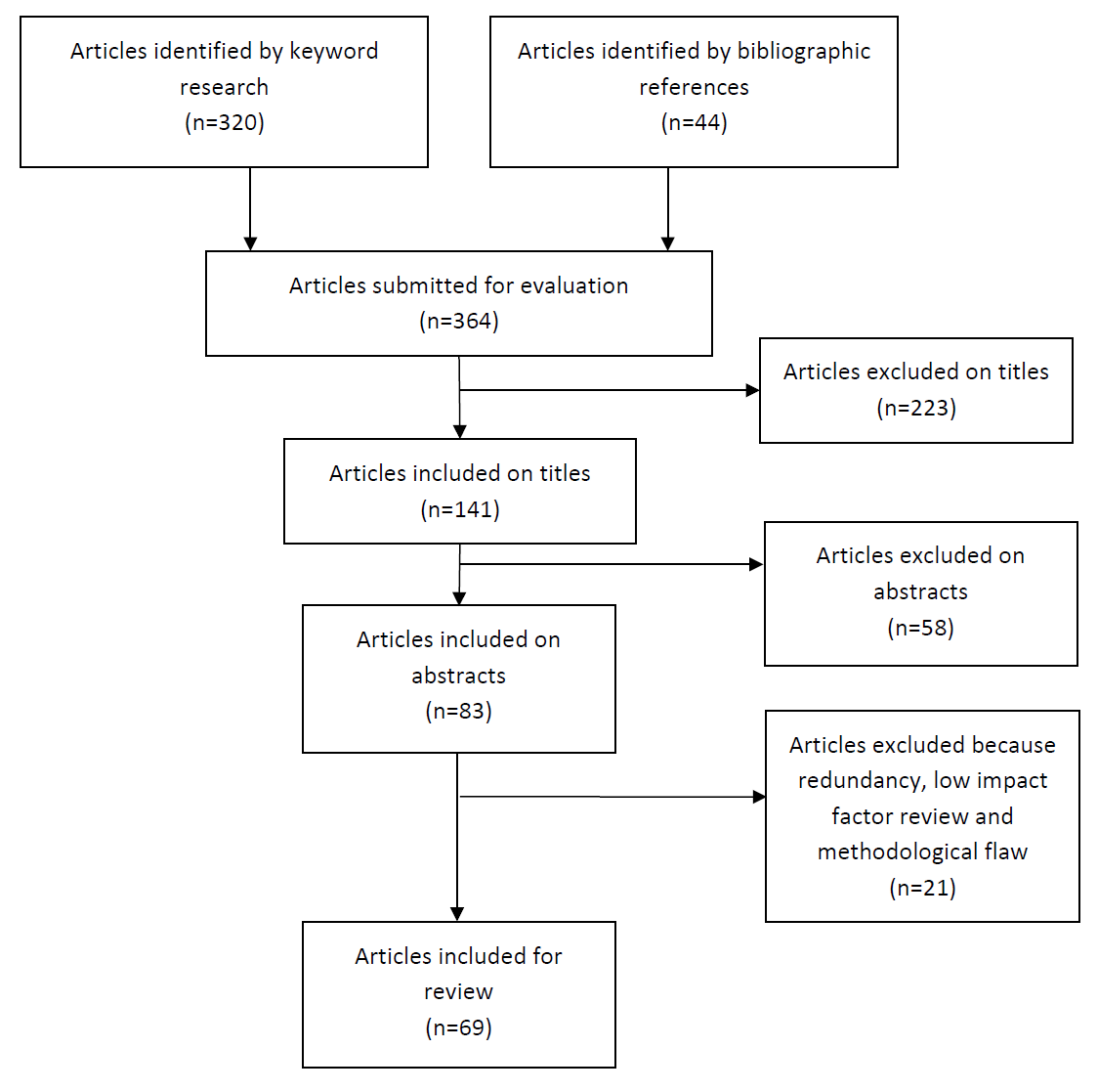
Preferred Reporting Items for Systematic Reviews and Meta-Analyses (PRISMA) diagram.

Two authors, AB and FF, separately screened 364 abstracts, and 62 articles were included. This narrative review was divided into 3 parts: assessment, intervention, and treatment outcome prediction. See the Preferred Reporting Items for Systematic Reviews and Meta-Analyses (PRISMA) diagram in Figure 1.

This paper is not about a study that included patients. It was therefore not submitted to an ethics committee.

## Results

Given the fact that those technologies were at different stages of development, we present them from the most developed to the least developed.

### Assessment

Three new assessment methods emerged from our review: electronic health (eHealth)-based assessment (using smartphone or internet apps), VR, and ML classification.

#### Electronic Health–Based Assessment

Smartphone assessment apps can either use a digital version of a validated scale or an ecological momentary assessment (EMA). Several apps are available or under development, but no study has so far validated assessment apps for OCD, and the use of Y-BOCS apps is not yet supported by research evidence [15]. The administration of psychological questionnaires via the internet is another form of assessment that may reduce the burden on patients and providers. These can be used for screening or for monitoring symptoms. The Web Screening Questionnaire is based on the Y-BOCS and can be completed in 2 min. It was found to have good sensitivity and adequate specificity for the detection of OCD symptoms when compared with a full-length structured clinical interview [16,17]. OCD can be identified using a Web-based test that mimics a structured interview: the Web-Based Depression and Anxiety Test has adequate sensitivity for OCD diagnosis (0.71) and good specificity (0.97), when compared with the Structured Clinical Interview for Diagnostic and Statistical Manual Mental Disorders [18]. Furthermore, the electronic Psychological Assessment System, a Web-based, self-report, multidisorder, clinical assessment and referral system showed a fair agreement with the clinical interview for OCD (kappa=0.39) [19]

With an EMA, patients respond to a series of questions based on their reported symptoms. This evaluation of symptoms daily in the patient’s usual environment is theoretically free from recall biases, as the patient self-assesses “right then, not later; right there, not elsewhere” [20]. This new method using active data (supplied by the patient) profoundly modifies the assessment procedure, by introducing a computerized third party between a doctor and patient. The use of dedicated smartphone apps allows patients to keep an accurate diary of their symptoms and behaviors. In a clinical case study of EMA use in 3 adults diagnosed with OCD, Tilley and Rees [21] compared the numbers and types of obsessions and compulsions captured using the Y-BOCS, compared with an SMS-based EMA. Participants were told to record their experiences across a 12-hour period, in reply to text message prompts. The EMA approach yielded a lower number of obsessive and compulsive symptoms but produced additional types of obsessions and compulsions that had not been identified before by the Y-BOCS. The authors concluded that the EMA‑OCD procedure may represent a worthy addition to the suite of assessment tools but requires research with larger samples.

#### Virtual Reality–Based Assessment

There are many definitions of VR, the most common being that it “refers to immersive, interactive, multi-sensory, viewer-centered, three-dimensional computer-generated environments and the combination of technologies required to build these environments” [22]. There is growing interest in VR as a key tool for investigating and assessing psychiatric disorders, as shown by the number of scientific articles with the term *virtual reality* in their title or abstract that are published in MEDLINE every year on this topic, which increased from 5 in 1991 to 842 in 2017.

Until recently, VR was limited by its cost and by the quality of the multimedia display technology. The recent democratization of these systems (Oculus Rift, PlayStation VR, HTC Vive, Samsung Gear) means that VR can now be used to perform neuropsychological assessments in real time [23]. The environment and the perceptual stimuli can be manipulated to trigger pathological behaviors (eg, checking behavior). This allows the clinician to assess behavioral responses to a situation that can elicit distress and to train patients how to cope with their symptoms.

Most research has focused on environmental trigger disorders (anxiety disorders in particular) [24], and we only found 3 studies of VR-based OCD assessment. This is difficult to understand, as this disorder seems an ideal candidate for this method, insofar as it is characterized by obsessions that are regularly induced by environmental triggers. A research group showed that the degree of VR-generated anxiety of individuals with OCD is positively correlated with their symptom and immersive tendency scores, suggesting that VR technology could be a useful anxiety-provoking tool [25]. When the same team replicated their study, they demonstrated that patients with OCD had significantly greater problems with compulsive checking than controls in a VR environment. Their results support the use of VR as a possible new behavioral measure of compulsive checking behavior [26].

Van Bennekom et al created a VR game from a first-person perspective that allowed patients to walk through a house where 15 OCD-related items were displayed, while simultaneously measuring their OCD symptoms (compulsions, anxiety, tension, uncertainty, and urge to control). A pilot study comparing 8 patients with OCD and 8 healthy controls showed that VR is capable of triggering OCD symptoms in patients, allowing clinicians to directly observe and assess OCD symptoms [27].

#### Machine Learning–Based Assessment

ML is the scientific discipline that focuses on how computers learn from data, using statistics to find relationships between them and efficient computing algorithms to accurately detect classification patterns [28]. This form of AI uses 2 different kinds of classification process: supervised and unsupervised. The former identifies rules from databases containing cases that have already been validated, while the latter looks for patterns in unlabeled data to find new structures. The coupling of ML with magnetic resonance imaging (MRI), electroencephalogram (EEG), or even blood tests can reveal patterns that allow patients to be divided into different groups (eg, patients at risk of relapse or patients with active disease). For example, ML is already showing considerable promise for predicting psychotic transition in patients in an at-risk mental state [29], and in the field of mood disorders [30].

We identified 5 studies in OCD, of which 3 were related to neuroanatomical data, 1 to EEG, and 1 to a set of both clinical and imaging data.

Hoexter et al applied support vector regression (SVR) to cortical volumes in individual structural MRI datasets of patients with OCD to predict symptom severity by identifying neurobiological markers. They found that the left medial orbitofrontal cortex (OFC) and left putamen contained the most discriminative information. Pearson correlation coefficient between predicted (with SVR) and observed (with Y-BOCS) symptom severity scores was 0.44 (*P*=.006), which is considered to be moderately positive [31].

Another study analyzed cortical and subcortical structures from MRI data of 38 patients with OCD and 36 controls and used different ML algorithms either without or with a feature selection algorithm (which is the process of selecting a subset of relevant features such as variables or predictors for use in model construction) to achieve an accurate distinction between patients and controls. Classification accuracy ranged from 52.56% (no feature selection) to 71.64% (*t* test with feature selection) [32].

Hu et al studied the application of multivariate pattern analysis to high-resolution T1-weighted MRI images acquired from 33 patients with OCD and 33 controls. The highest classification accuracy (81.82%) was achieved by a support vector machine (SVM) classifier using white matter information [33].

ML-based EEG classification is gaining interest for several psychiatric disorders, as it has very high classification accuracy among mood disorders, up to 98.95% classification accuracy for bipolar disorder versus schizophrenia [34], and 80.19% for unipolar versus bipolar depressive disorder [30]. High classification accuracy has also been found for OCD. Examining single-channel EEG and 2-channel interhemispheric dependency measurements to distinguish between patients with OCD (n=10) and controls (n=10), using SVM classifiers, resulted in 85% (SD 5.2) classification accuracy [35].

Mas et al applied 2 supervised classification methods of ML-based class prediction to a dataset that included structural MRI, diffusion tensor imaging, neuropsychological, and genetic (single-nucleotide polymorphisms) data to predict early onset OCD severity. Their model classified child and adolescent patients with OCD by disease severity with an accuracy of 90% in the test set and 70% in the validation sample [36].

### Interventions

Many treatments are offered in daily practice, but only 2 have been scientifically validated for OCD: pharmacotherapy (antidepressant) and CBT, including exposure and response prevention (ERP). It is easy to digitally transpose CBT using personalized modular programs in a smartphone app, on the internet, or in a VR program (for a review see Aboujaoude [37]). Technologies can also be used to augment traditional CBT [38,39], and could address issues of accessibility and effectiveness by increasing treatment adherence. Some authors refer to this type of treatment as *tCBT*, for technology-empowered CBT, or *cCBT*, for computerized CBT [40]. A review assessing the comparative efficacy of tCBT versus therapist-administered CBT (TA-CBT) found that tCBT and TA-CBT did not differ significantly in their efficacy on OCD symptoms, although there was a trend favoring TA-CBT [41]. Their study used mixed different technologies (self-help books, leaflets, bibliotherapy, internet, webcams, telephones, phone-delivered interactive voice response systems, and CD-ROMs), thereby making it impossible to disentangle the specific effects of the new technologies (smartphone in particular) delivering CBT. In addition, biofeedback, a new type of treatment using a computer, is starting to emerge, but was not included in any of the previous reviews [37].

Articles were divided in 5 categories: smartphone-based, Web-based (online program) or computer-based (ie, software) interventions, VR, and biofeedback. These categories were chosen because they correspond to the most innovative topics and are most often reported in studies of new technologies in psychiatry.

#### Telemedicine-Based Interventions

We found 9 studies, 2 case series reports, and 6 trials. All trials concluded that the videoconference intervention was effective and very acceptable for patients with OCD. However, face-to-face control design studies are lacking. One open-label trial concluded that a videoconferencing-based intervention is effective in the treatment of OCD in adults [42]. Among 5 randomized trials, 2 focused on the young with OCD [43,44], with 1 trial having control waitlist, and 1 trial having control consisting of face-to-face family-based CBT. The results are summarized in Table 1.

**Table 1:** Videoconferencing-based interventions.

Study, year	Aim	Methods	Results
Storch et al [43], 2011	To assess the efficacy of a 12-week webcam-delivered CBT^a^ with therapist (14 sessions of 60‑90 min) plus a brief initial face-to-face session in young OCDs^b^.	31 youth with OCD (range=7‑16 years; 19 male) were randomly assigned to webcam-delivered CBT or a waitlist control. Assessments were conducted immediately before and after treatment, and at a 3-month follow-up (for webcam-delivered CBT arm only). Primary outcomes included CY-BOCS^c^, CGI^d^ rates, and remission status.	Webcam-delivered CBT was superior to the waitlist control on all primary outcome measures with large effect sizes (Cohen *d*≥1.36) and at follow-up within group (Cohen *d*≥1.98). In all, 13 of 16 participants of the treatment group reached at least 30% CY-BOCS reduction and were considered as responders.
Goetter et al [42], 2014	To assess the efficacy of videoconference-mediated (16‑18 twice-weekly, 90-min, individual sessions with between-session phone check-ins), in adult OCDs.	Open trial involving 15 participants. Assessment at four different times: pretreatment, midtreatment, posttreatment, and 3‑month follow-up. Primary outcomes included Y-BOCS^e^, CGI rate and Quality of Life Enjoyment and Satisfaction Questionnaire short form score.	The pre and post effect sizes were significant for the OCD symptom severity (Hedges *g*=2.56) and quality of life (Hedges *g*=1.27), and 80% of participants were rated as very much or much improved on the CGI. A total of 30% participants no longer met DSM-IV-TR^f^ criteria for OCD among the 10 individuals who completed the 3-month follow-up assessment.
Vogel et al [45], 2014	To assess the efficacy and working alliances of technology along with telephone calls in adult OCDs: 6 tablet-based videoconferencing sessions (N=6) or studio-based videoconference (N=4), and 9 telephone sessions.	30 adults were randomized to 12-week videoconference-assisted ERP^g^ (N=10), self-help ERP (N=10), or a waitlist condition (N=10). Primary outcome included Y-BOCS score, and Working Alliance Inventory. Assessments were conducted before and after treatment (12 weeks) by a psychologist blinded to treatment condition.	Videoconferencing treatment produced significantly greater reductions in obsessive-compulsive symptoms compared with the 2 control conditions (post hoc analysis videoconferencing treatment compared with self-help, *P*=.01, and waitlist, *P*=.01). Patients rated the videoconferencing format as natural and reported strong working alliances with their therapists.
Herbst et al [46], 2014	To assess the efficacy of a Web-based writing therapy with therapeutic interaction based on the concept of CBT (iCBT^h^; 8-week treatment of 14 sessions) for adult OCD.	34 adults were randomized according to a waitlist control design with follow-up measures at 8 weeks and 6 months. The primary outcome was the change in the severity of OCD symptoms (Y-BOCS, self-report, and Obsessive-Compulsive Inventory-Revised OCI-R).	iCBT treatment produced significantly greater reductions in obsessive-compulsive symptoms compared with the waitlist control group (Cohen *d*=0.82 Y-BOCS SR and *d*=0.87 OCI-R), using an intention-to-treat analysis. This effect remained stable at the 6-month follow-up. Of the 30 completers, 90% rated their condition as improved and would recommend the program to their friends.
Herbst et al [47], 2016	To assess the patient-therapist relation of a Web-based writing therapy with therapist guidance based on the concept of CBT (iCBT; 8-week treatment of 14 sessions) for adult OCD.	30 adults were randomized according to a waitlist control design with follow-up measures at 8 weeks. Primary outcome focused on Working Alliance Inventory self-report posttreatment within group.	The posttreatment Working Alliance Inventory-SR composite score represented 77% of the maximum scale value, which indicates a high working alliance.
Comer et al [44], 2017	To assess the working alliance and treatment satisfaction of videoconferencing in early OCD youth.	22 young patients with OCD (aged between 4-8 years) were randomized into videoconference-delivered family-based CBT or clinic-based family-based CBT. Pre-and posttreatment, and 6-month follow-up assessments masked to treatment condition. Primary outcomes were scores on working alliance and treatment satisfaction (Client Satisfaction Questionnaire).	Treatment alliance and satisfaction were high across conditions. At posttreatment, 72.7% of internet cases and 60% of clinic cases showed “excellent response,” and at follow-up, 80% of internet cases and 66.7% of clinic cases showed “excellent response” (defined as a 1 or 2 on the CGI Scale). Differences between conditions on clinical significance responder status were nonsignificant.

^a^CBT: cognitive behavioral therapy.

^b^OCD: obsessive-compulsive disorder.

^c^CY-BOCS: Children’s Yale‑Brown Obsessive‑Compulsive Scale.

^d^CGI: Clinical Global Impression Scale.

^e^Y-BOCS: Yale‑Brown Obsessive‑Compulsive Scale.

^f^DSM-IV-TR: Diagnostic and Statistical Manual of Mental Disorders fourth edition.

^g^ERP: exposure and response prevention.

^h^iCBT: Web-based cognitive behavioral therapy.

#### Smartphone-Based Interventions

A recent review analyzed the current status of eHealth apps in the field of depressive and anxiety disorders, including OCD [15]. The authors highlighted the lack of a smartphone app in this field for intervention, as they only found one study—a case report that discussed the utility of a smartphone-based geofeedback app for patients with excessive outdoor checking behavior. This app delivers an audio message (recorded alarm signal) if a compulsive behavior is suspected (ie, if the patient takes too long to cover a predefined distance) [48]. The goal of reducing the time taken to get to appointments was reached (from 2 hours down to 20 min), not because of the app itself, but because the patient did not want to attract the attention of people around him.

In the wake of this review, an open pilot trial investigated the feasibility, acceptability, and efficacy of an ERP app (LiveOCDFree) among 21 patients with mild to moderate symptoms [49]. This showed that smartphone-guided ERP is feasible and acceptable, with high rates of retention and satisfaction, as participants reported significant improvements in OCD symptoms, showing 4.25-point improvement on self-report Y-BOCS (*F*_2,40_=4.25, *P*=.02), and 3.96-point improvement on Beck Anxiety Inventory (*F*_1.31,26.13_=3.96, *P*=.047), although not in depressive symptoms or quality of life. However, symptom amelioration only occurred within the first 6 weeks of this treatment, and there was no further improvement after midtreatment.

#### Web-Based Interventions

We found 19 studies on this topic. Most concluded that a Web-based treatment is effective, highly acceptable to patients with OCD [50], and may reduce barriers to treatment access [51]. The results are summarized in Multimedia Appendix 1 [52-62].

Regarding the patient‑therapist relationship, a systematic review found no differences between Web-based CBT (iCBT) and face-to-face therapy [63].

#### Computer-Based Interventions

Few studies have specifically focused on computerized CBT for OCD, of which 6 [64-69] featured computer-driven telephone interview system (BT Steps). Y-BOCS effect sizes (mean Cohen *d*=0.84) were smaller than those for therapist-led CBT (mean Cohen *d*=1.22) [70]. The most recent study [68] randomly assigned 87 patients to 12 weeks of treatment with either BT Steps alone (n=28), BT Steps with nontherapist coaching (n=28), or BT Steps with CBT therapist coaching (n=31). All 3 interventions brought about a significant reduction in Y-BOCS scores, with effect sizes (Cohen *d*) of 1.16, 1.41, and 1.12, respectively. The main finding of this study was that when patients were asked which method of therapy (computer vs clinician) they preferred, they chose iCBT (computer: 48%; face-to-face therapy: 33%; and no stated preference: 19%). For a review see Lovell and Bee [71]. It should be noted that BT Steps was subsequently modified to be used online, and its name was changed to *OCFighter* [57], but Lovell et al found that, when used with low intensity, OCFighter does not lead to clinically significant benefits, although it may reduce uptake of therapist-led CBT [72].

In another study, patients underwent three 45-min sessions at weekly intervals on an interactive computer program that provided vicarious exposure and response prevention for OCD, but no significant change in Y-BOCS scores was reported, although a significant change in depressive symptoms was observed [73].

One study focused on a computerized psychoeducative tool as an add-on to standard CBT, reporting variable acceptance across patients and no difference in the Y-BOCS score reduction when compared with a group using standard CBT alone [74].

Kalanthroff et al [75] developed a program called *Personalized-Computerized Inhibitory Training* (P-CIT) that sought to improve patients’ ability to inhibit responses when exposed to images that were related to their specific OCD symptoms. They combined P-CIT and ERP in an 11-patient study involving training with P-CIT in 3 sessions of 15 min for 7 consecutive days, followed by 8 in-person 60-min ERP sessions with a trained therapist delivered over 2 or 3 weeks, all the while continuing P-CIT. The Y-BOCS score change was estimated over time, at weeks 0, 1, and 3. Y-BOCS scores decreased significantly over time (b=−3.47/week, *t*_19_=−7.46, *P*<.001), and all patients save one achieved remission, with a Y-BOCS reduction of at least a 35% (mean reduction of 11 points).

#### Virtual Reality–Based Interventions

Kim et al [76] used a virtual environment to produce variations in arrangement anxiety in 24 patients with compulsive arranging symptoms. Patients performed virtual arrangement tasks 3 times, at 3-day intervals, and results showed that arrangement-related anxiety levels decreased significantly between the first and last days. No Y-BOCS scoring was used in this study.

Laforest et al [77] enrolled 3 adults with contamination OCD and exposed them to 2 virtual environments: a training environment (*neutral*) and an experimental (*contaminated*) environment. They assessed the presence and intensity of obsessions and compulsions (baseline, 3, 4, and 5 weeks, and at the end of a 12-session treatment). Exposure in VR (ie, touching walls and toilet bowls with varying degrees of filthiness) was discussed during a CBT session (reviewing the exposure session, performing cognitive restructuring of dysfunctional thoughts, and discussing upcoming homework assignments). The authors found a significant improvement in all 3 participants: pretreatment Y-BOCS scores were 22, 31, and 30, respectively, and at the 4-month follow-up were 16, 11, and 23, respectively. It should be noted that at the 8-month follow-up, Patient 2 still had a reduced Y-BOCS score, but the Y-BOCS scores of the other 2 patients had risen to 21 and 27.

#### Biofeedback-Based Interventions

Developed in the 1970s [78], biofeedback is a painless, noninvasive procedure that consists of capturing biometric data such as EEG, ECG, electromyogram (EMG), skin conductance, and temperature, and immediately feeding them back to the patient. The objective is to model the patient’s brain activity in real time as an image (video game type) or sound. Based on CBT techniques and relaxation, patients gradually learn to promote brain activity corresponding to the therapeutic target, through positive reinforcement. When activity in a desirable frequency band increases, the symbol modeling the brain activity changes in one direction, and when activity in an unfavorable band increases, the symbol changes in the opposite direction. Patients gradually learn the new brainwave, taking a wave corresponding to what is observed in healthy individuals as their model.

The first use of biofeedback in the field of OCD goes back to 1977, when a 25-year-old woman was treated with systematic desensitization in which EMG biofeedback was used to achieve relaxation [79].

Sürmeli and Ertem used quantitative EEG-guided neurofeedback (NF) in a case series of 36 treatment-resistant patients with OCD. The NF intervention consisted of inhibiting (keeping the activity below a set threshold) EEG theta or alpha rhythms on frontal, prefrontal and frontotemporal deviations. All participants underwent daily 60-min sessions for 9 to 84 days. Results showed that 91% of participants who received NF training showed a clinical improvement, according to the Y-BOCS and Clinical Global Impression (CGI) Scale, and 52% of participants maintained the improvements in their OCD symptoms at the 26-month follow-up [80].

Another study assessed NF efficacy in a randomized, double-blind, parallel design, involving 20 inpatients with OCD who underwent 25 sessions of either NF or sham (placebo) feedback (SF). The aim of the NF intervention was to reduce EEG activity in an independent component previously reported to be abnormal for this diagnosis. Although a pre- versus posttreatment comparison of the trained component and frequency did not yield significant results, the NF group had a significantly greater reduction in compulsions, compared with the SF group (*P*=.015) [81].

Deng et al used EEG biofeedback training as an adjunct to standardized treatment (antidepressant medication plus CBT) in a randomized controlled trial involving 79 patients with OCD. Of these, 40 were randomly assigned to the study group (antidepressant medication plus 8-week CBT plus NF sessions 5 times/week), and 39 were randomly assigned to the control group (antidepressant medication plus 8-week CBT). At 8 weeks, treatment was considered effective in 86.5% of participants in the study group and in 62.9% of participants in the control group, with mean decreases in the Y-BOCS score of 14.44 (study group) and 13.2 (control group)—a statistically significant (*P*=.003) but clinically irrelevant difference [82].

#### Machine Learning–Based Prediction

Five studies investigated whether ML approaches can predict treatment response or symptom severity.

Salomoni et al studied 130 participants under pharmacotherapy with selective serotonin reuptake inhibitors (SSRI, alone or SSRI plus low-dose risperidone) and/or CBT (ERP mostly), using 3 variables (Y-BOCS symptoms dimension, neuropsychological performances, and epidemiological data) to predict treatment outcome at 3 or 6 months. When a multilayer perceptron (ie, supervised artificial neural network) was compared with a classic logistic regression model, it was found to have a considerably better predictive performance (93.3% vs 61.5%), when it came to correctly classifying patients as nonresponders to treatment (46.9% of participants) [83].

Another team used SVR to assess whether structural MRI assessing volumetric brain matter could predict symptom severity in 37 patients with no prior treatment. They found weak Pearson correlation coefficients (0.44‑0.49) between observed and predicted severity using the Y-BOCS and dimensional Y-BOCS. The main value of this study was to highlight the ability of ML to identify neurobiological markers of OCD, as some regions contained more discriminative information than others: the left medial OFC) and left putamen were associated with severity, while the best predictors of the sexual/religious OCD subtype were the left medial OFC, right lateral OFC, and left anterior cingulate cortex [31].

Yun et al also used structural MRI to individualize biomarkers (cortical morphology) of treatment response to SSRI-based pharmacotherapy using SVM. A total of 56 treatment-naive patients with OCD and 75 healthy controls underwent T1-weighted MRI at baseline and after 4 months of newly introduced SSRI treatment (patients only). The SVM algorithm correctly classified the responders and nonresponders (based on variation in Y-BOCS score) with 90.7% to 95.6% accuracy (sensitivity=90.8%‑96.2%; specificity=91.1%‑95%) [84].

Askland et al used several variables (including Y-BOCS items, Neuroticism, Extraversion, and Openness to Experience-Five Factor Inventory items and subscale scores, Y-BOCS symptom checklist cleaning/washing compulsion score, and several self-report items from social adjustment scales), implemented in a Random Forest ML algorithm to predict remission outcome. Using 26 high-confidence features to predict a binary outcome (remitted vs never remitted), the algorithm correctly classified the patients (N=296) in 76.18% of cases (error rate=23.82%; bootstrap CI 22.10‑25.45) [85].

Lenhard et al tested the ability of 4 different ML methods to predict treatment response to iCBT in a sample of 61 adolescents (12‑17 years of age) with OCD. Participants were enrolled in a randomized controlled trial and received either immediate iCBT or delayed iCBT. The authors compared multivariate logistic regression with 4 ML algorithms (1 linear model with best subset predictor selection, and 3 flexible models: LASSO, Random Forest, and SVM) implemented with 46 demographic and clinical baseline variables (eg, Children’s Y-BOCS score, OCD onset, OCD duration, symptom dimension, and CGI). The multivariate logistic regression was unable to detect significant predictors, whereas the 4 ML algorithms allowed treatment response to be predicted with between 75% (LASSO, Random Forest, and SVM) and 83% (linear model with best subset predictor selection) accuracy [86].

## Discussion

### Principal Findings

There is a constantly growing body of knowledge in the field of OCD care, and it is becoming increasingly complicated to handle all these data on a daily basis. Our systematic review shows that the evaluation of new innovative technologies in the OCD is heterogeneous. Thus, the number of randomized studies available is low. With 5 randomized studies, telemedicine and the Web-based interventions are the most robustly evaluated.

In addition, more validated tools are needed to optimize the management of OCD. In the present review, we highlighted the diversity of new technologies used in psychiatry and their application to OCD for the purposes of prediction, diagnosis, and intervention. e-psychiatry is already booming, and some even talk about a digital mental health revolution [87]. These conclusions agree with the other reviews carried out in the field of psychiatric disorders (in particular PTSD and addictions) which highlight the interest of these new technologies [12,13].

### Strengths and Limitations

However, EMA provide additional data but do not replace passing a scale with a trained clinician, and the reviewed studies does not bring systematic information about EMAs sensitivity and specificity. The timing (screening, evolution) or frequency of their use is not well defined. In addition, the absence of external contributor does not make it possible to ensure the veracity of the data collected.

As we have seen, VR seems very promising in OCD but in practice, in vivo exposure can hardly be proposed to all patients, and so does virtual exposure. Furthermore, an important limitation concerns the possibility of customizing the environment and the device tolerance since some side effects (such as dizziness, nausea, headache, and eyestrain) are not enough evaluated. As for the EMA, the optimal therapeutic protocol is not yet clearly defined (duration, number of sessions) as well as the duration in time of the effect, or the use in children. CBT supported by smartphone, internet, or computer clearly offers new therapeutic opportunities, but they may suffer from a lack of human interaction with more uncertain adherence (therapeutic alliance). The impossibility of finely adjusting coping strategies to progress is also a shortcoming. The question of self-administration of a treatment is also a significant risk. These risks seem lower with telemedicine, an older practice that also benefits from recent technological developments. It also provides solutions to the difficulties of access to care. Interaction with a health professional reduces the risk of poor compliance and allows better therapeutic adaptation.

In the end, few data are available on the severe forms of the disorder, classically excluded from studies.

The acceptability of these technologies must therefore be assessed at different levels [14,88]. This assessment is generally based on several major criteria: usability (device’s flexibility and ease of learning), utility (technology’s contribution), and satisfaction and reliability (including accuracy, effectiveness, and efficiency). Cost, though fundamental, is a secondary consideration. Finally, the concept of risk impinges on acceptability and constitutes an important dimension of medical reasoning. It must therefore be taken into account when these technologies are being assessed (impact of false positives or false negatives, ethical issues).

In addition to the concerns of acceptability, validity, cost, and data security, it is necessary to consider the specific problems of patients with OCD. The major component of this disorder is the verification ritual, which is performed to relieve the anxiety associated with obsessive ideas and intrusive doubt [89]. Even if this is not reported in studies, care should be taken to ensure that users do not develop digital stress or become a slave to an app or VR software.

The data on detection may not have the same impact as in other areas of psychiatry, in which delayed diagnoses are more common (eg, mood disorder or psychotic transition). However, new forms of assessment (EMA, VR- or ML-based assessment) perform better than standardized tests, and sometimes even better than therapist interviews.

The data that are currently available indicate that these new technologies could be extremely valuable in the field of treatment. Although they are not necessarily more effective than qualified practitioners, these new tools allow for the democratization of access to recommended therapies, including CBT and iCBT (via smartphone, internet, or computer), are easy to use, well accepted by patients, reproducible, and cost-effective. Enhanced CBT with VR makes exposure therapy possible without moving out of the clinical setting but needs a high degree of personalization to be effective. Furthermore, the VR could trigger the stimulus-response OCD pattern of the patient, and therefore, if not enough guided by a trained therapist, it could be a risk needed to be considered. NF, which is in an early stage development compared with other interventions, has however proved to be useful in reducing compulsion in some patients. Finally, in the field of prediction, severity or treatment success can be predicted with a good degree of accuracy. Being able to predict the response to a treatment will promote personalized medicine. Furthermore, given the current development of new technology in the psychiatric field, it seems important to increase training measures on these technologies, particularly by integrating them into the resident teaching program. Many ethical issues, data security, data storage, privacy, and hacking risk have yet to be resolved. Disease detection or risk prediction of OCD could be stressful for patients and brings the risk of excessive focus and anxious counter reaction. It is essential for psychiatrists to be involved in the development of these technologies, and developers have a major interest in communicating better about the design of these tools and the algorithms they want us to use in the near future.

### Conclusions

Confidence in eHealth among patients with addictions and health care professionals is a major issue [90]. Studies have highlighted good acceptability and patient compliance. ML is revolutionizing fundamental research, by allowing for better classification of patients, based not only on clinical data but also on biological or neuroimaging-derived data. It is becoming reasonable to talk about genuinely complementary examinations in behavioral studies. Finally, these new technologies are enabling the development of new therapies, including biofeedback and VR, that focus on the learning of coping skills.

## Appendix

Multimedia Appendix 1 Web-based intervention studies.

## Abbreviations

AI: artificial intelligence
BT Steps: computer-driven telephone interview system
CBT: cognitive behavioral therapy
cCBT: computerized cognitive behavioral therapy
CGI: Clinical Global Impression scale
CY-BOCS: Children’s Yale‑Brown Obsessive‑Compulsive Scale
ECG: electrocardiogram
EEG: electroencephalogram
eHealth: electronic health
EMA: ecological momentary assessment
EMG: electromyogram
ERP: exposure and response prevention
iCBT: Web-based cognitive behavioral therapy
ML: machine learning
MRI: magnetic resonance imaging
NF: neurofeedback
OCD: obsessive-compulsive disorder
OFC: orbitofrontal cortex
PRISMA: Preferred Reporting Items for Systematic Reviews and Meta-Analyses
PTSD: posttraumatic stress disorder
SF: sham feedback
SSRI: selective serotonin reuptake inhibitors
SVM: support vector machine
SVR: support vector regression
TA-CBT: therapist-administered cognitive behavioral therapy
tCBT: technology empowered cognitive behavioral therapy
VR: virtual reality
Y-BOCS: Yale‑Brown Obsessive‑Compulsive Scale
