# Supplementary material for: How New Technologies Can Improve Prediction, Assessment, and Intervention in Obsessive-Compulsive Disorder (e-OCD): Review
Source: JMIR Ment Health. 2019 Dec 10;6(12):e11643. doi: 10.2196/11643 (PMC6930507; doi:10.2196/11643)
Supplement: Multimedia Appendix 1 [file mental_v6i12e11643_app1.docx]

Multimedia Appendix. Web-based intervention studies.

| Study, year | Aim | Methods | Results |
| --- | --- | --- | --- |
| Andersson et al [41], 2011 | Assess the efficacy of a 15-week Web-based CBT^a^ (iCBT^b^) program with therapist support (psychoeducation, cognitive restructuring and ERP^c^). | Open trial with 23 patients. The primary outcome was the Y-BOCS^d^ score. | 61% of participants had a clinically significant improvement and 43% no longer fulfilled the diagnostic criteria for OCD^e^ at the end of the intervention, with a large within-group effect size (Cohen *d*) of 1.56. |
| Wootton et al [42], 2011 | Assess the efficacy of an iCBT with 8 online lessons delivered over 8 weeks and incorporating cognitive and behavioral techniques. | Open trial with 22 patients. The primary outcome was the Y-BOCS score. | 81% of participants completed the program. Participants improved significantly (39.72% reduction in Y-BOCS score) with within-group effect size (Cohen *d*) at follow-up of 1.28. |
| Andersson et al [43], 2012 | Assess the efficacy of a 10-week iCBT program compared with an attention control condition (online supportive therapy). | Randomized controlled trial with 101 patients. The primary outcome was the Y-BOCS score. | iCBT resulted in greater improvements than the control condition, with a significant between-group effect size (Cohen *d*) of 1.12 posttreatment. The proportion of participants showing a clinically significant improvement was 60% for the iCBT group, compared with 6% for the control condition. |
| Herbst et al [44], 2014 | Assess the efficacy of 14 sessions of internet-based writing therapy with therapeutic interaction based on CBT with ERP. | 34 patients were randomized to receive the intervention either directly or with an 8-week delay. The primary outcome was the Y-BOCS self-report score. | OCD symptoms were significantly improved in the treatment group, compared with the waiting-list control group, with large effect sizes (Cohen *d*) of 0.82 posttreatment (and stable at the 6-month follow-up). |
| Lenhard et al [45], 2014 | Assess the efficacy and acceptability of a 12-week iCBT platform (BiP OCD) with therapist support for adolescents. | Open trial with 21 patients (12‑17 years). The primary outcome measure was the CY-BOCS^f^. | OCD symptoms improved significantly, with a large effect size (Cohen *d*) of 2.29. At the 6-month follow-up, 71% were classified as responders (≥35% decrease in CY-BOCS score), and 76% as being in remission (CY-BOCS score ≤ 12). |
| Andersson et al [46], 2014 | Assess the long-term efficacy of iCBT with therapist support with or without a Web-based booster program (self-help text with worksheets and an integrated email system). This was a continuation of the study described above [42]. | 93 of the 101 patients in the previous study were randomized to either a booster program or no booster program. The primary outcome was the Y-BOCS score. | The booster group had a significant mean reduction in OCD symptoms, compared with the control condition, from booster baseline (4 months) to 7 months, *F*_83,35_=5.951, *P*<0.05, but not to 12 or 24 months. |
| Mahoney et al [47], 2014 | Assess the efficacy of 6 online lessons, completed over 10 weeks, of a technician-administered iCBT (including ERP and psychoeducation), compared with a treatment-as-usual control group. | 67 patients were randomized to either iCBT or treatment-as-usual. The primary outcomes were scores on the Dimensional Obsessive‑Compulsive Scale and Obsessional Beliefs Questionnaire. | The iCBT program was more effective than treatment-as-usual in reducing maladaptive obsessive-compulsive beliefs, as well as OCD symptoms, with large within- and between-groups effect sizes (>78) revealed by a linear mixed model ANOVA. |
| Diefenbach et al [48], 2015 | Assess the efficacy of a 17-week internet-guided self-help program (OCFighter) involving 9 treatment steps (psychoeducation, planning, etc). | Open trial with 24 patients. The primary outcome was the Y-BOCS score. | The intent-to-treat sample exhibited statistically significant improvements with a large effect size (Cohen *d*) of 0.87 at posttreatment. |
| Rees et al [49], 2016 | Assess the efficacy of an 8-stage, completely self-guided iCBT treatment based on ERP (OCD? Not Me!) for adolescents. | Open trial with 132 patients (12‑18 years). The primary outcome was the score on the self-report version of the Children’s Florida Obsessive-Compulsive Inventory. | Preliminary results showed significant reductions in OCD symptoms, *F*_8,285_=7.38, *p*<001, with a moderate effect size (Cohen *d*) of 0.64 at posttreatment. |
| Seol et al [50], 2016 | Assess the efficacy of an 11-session iCBT program involving ERP, psychoeducation, belief restructuring and relapse prevention, in a Korean clinical setting. | Open trial with 57 patients. The primary outcome was the Y-BOCS score. | 42 patients completed all the training sessions, and 15 were classified as non-completers. OCD symptoms were significantly improved, with a large effect size (Cohen *d*) of 1.68. |
| Lenhard et al [51], 2016 | Assess the efficacy of 12-week therapist-guided iCBT for adolescents (BiP OCD), compared with a waitlist control condition. This was an extension of the study described above [44]. | Randomized controlled trial with 67 patients (12‑17 years). The primary outcome measure was the CY-BOCS score. | In intention-to-treat analyses, intervention was superior to waitlist, with a moderate effect size (Cohen *d*) of 0.69 posttreatment. At the 3-month follow-up, patients randomized to BiP OCD showed further improvement, with a within-group pretreatment to follow-up effect size (Cohen *d*) of 1.68. Patient satisfaction with BiP OCD was high. |
| Lenhard et al [52], 2017 | Evaluate the cost-effectiveness of therapist-guided iCBT for adolescents (BiP OCD) compared with a waitlist control condition. This was an extension of the study described above [50]. | Cost data (health care use, supportive resources, prescription drugs, prescription-free drugs, school absence, productivity loss, and cost of iCBT) were collected at baseline and after treatment. Bootstrapped mixed model analyses were conducted, comparing incremental costs and health outcomes between the groups. | iCBT generated substantial societal cost savings averaging US $ 144.98 (95% CI −159.79 to −130.16]) per patient. iCBT was a cost-effective treatment compared with the condition where patients did not receive evidence-based treatment. |

^a^CBT: cognitive behavioral therapy.

^b^iCBT: Web-based cognitive behavioral therapy.

^c^ERP: exposure and response prevention.

^d^Y-BOCS: Yale‑Brown Obsessive‑Compulsive Scale.

^e^OCD: obsessive-compulsive disorder.

^f^CY-BOCS: Children’s Yale‑Brown Obsessive‑Compulsive Scale.
